# Lyotropy as a Design Consideration for Ultra‐Small Protein Nanoparticles via Electrohydrodynamic Jetting

**DOI:** 10.1002/marc.202500533

**Published:** 2025-08-14

**Authors:** Muhammad Haseeb Iqbal, Julio Zelaya, Quy Ong, Francesco Stellacci, Joerg Lahann

**Affiliations:** ^1^ Institute of Functional Interfaces Karlsruhe Institute of Technology Karlsruhe Germany; ^2^ Biointerfaces Institute University of Michigan Ann Arbor Michigan USA; ^3^ Institute of Materials Ecole Polytechnique Fédérale de Lausanne (EPFL) Lausanne Switzerland; ^4^ Bioengineering Institute Ecole Polytechnique Fédérale de Lausanne (EPFL) Lausanne Switzerland

**Keywords:** electrospraying, hofmeister effect, nanoparticle size, proteins, salts

## Abstract

Protein‐based nanoparticles offer tailored bioactivity and biodegradability that are distinct from their synthetic polymeric counterparts. Precise engineering of physical properties, especially size, of nanoparticles using electrohydrodynamic (EHD) jetting is a crucial factor that defines the fate of delivery systems in nanomedicine. Herein, we establish a systematic understanding that leads to the preparation of human serum albumin (HSA) nanoparticles with sizes as small as 50 nm. Interestingly, the addition of salt at very low concentrations, around 1–5 mm, combined with EHD process parameters, can result in narrow distributions of particle sizes that are consistently below 100 nm. At a given concentration, i.e., 2 mm, anions modulate the particle diameters that follow the Hofmeister Series as SO_4_^2−^ < CO_3_^2−^ < H_2_PO_4_^−^ < Cl^−^ < I^−^. This size reduction is primarily due to increased solution conductivity and interfacial charge density induced by salt ions during the EHD jetting process. High mobility ions compensate for the higher surface energy required to produce ultra‐small nanoparticles. Tight control over the size and distribution of ultra‐small nanoparticles may be critical for targeted drug delivery, as it can influence nanoparticle tropism or affect their cellular uptake.

## Graphical abstract

Lyotropic Ion‐Specific Effects Drive the Synthesis of Ultra‐Small Protein Nanoparticles via Electrohydrodynamic Jetting.

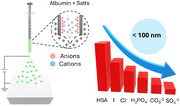

## 1 Introduction

Protein nanoparticles have garnered significant interest due to their growing applications in food and nanomedicine, thanks to their unique properties, besides innate protein characteristics, like the range of available proteins, conjugation capabilities, biodegradability, and low immunogenicity [1]. Besides numerous applications in food and processing industries, the potential of nanoparticles, particularly, as protein therapeutics has been significantly explored during the past two decades. Engineering the physical properties, especially the size and shape of nanoparticles, are crucial factors that determine their fate in biomedicine. Notably, the size of nanoparticles is known to dictate nanoparticles’ tropism, blood circulation time, mode of delivery, therapeutic loading efficiency, off‐target toxicity, and clearance pathways [2, 3, 4]. Nanoparticles are also actively subjected to clearance by the reticuloendothelial system (RES), which is influenced by their physicochemical properties. For instance, nanoparticles with diameters ≤5 nm undergo rapid renal filtration, whereas larger microparticles (>1 µm) tend to accumulate in organs such as the liver, spleen, and lungs [5]. For example, gold nanoparticles with a size of 50 nm showed better uptake by HeLa cells than those with 14, 30, 74, and 100 nm sizes [6]. Poly(lactide‐co‐glycolide) PLGA nanoparticles with an average size of 174 nm showed greater intralysosomal localization compared to the average sizes of 378 and 575 nm [3]. Increasing the size around or above 100 nm has been reported to delay RES clearance [7]. Fine control of the size and size distribution of the nanoparticles can thus be leveraged to improve therapeutics.

Among various methods to synthesize nanoparticles, electrohydrodynamic jetting (EHD) has emerged as a promising high‐throughput technique for producing synthetic protein nanoparticles (SPNPs) with micro‐ to nano‐scale diameters and narrow dispersity. This method enables the fabrication of versatile, multifunctional, and multicompartmental particles while preserving the secondary structure of proteins and allowing the loading of both hydrophobic and hydrophilic drugs [8, 9]. Various factors, e.g., electrical potential [10, 11], flow rate [12], collector distance [13], viscosity [14], solvent composition, addition of charged species like surfactants [3], surface tension, and electrical conductivity [15], have been exploited to influence the size of particles. These factors are often interconnected and can be modulated to fine‐tune the properties of the resulting SPNPs.

Free surface energy and charge‐energy compensation can explain limitations in making ultra‐small nanoparticles via EHD jetting. In EHD jetting, an electrical potential is applied to the tip of the needle that deforms the solution meniscus into a Taylor cone. Above a critical voltage, a continuous jet is ejected, breaking into droplets due to Rayleigh instabilities. Beyond the Rayleigh limit, the parent droplet undergoes Coulombic fission (as charge–charge repulsion overcomes surface tension), distributing the charge and reducing the electrostatic energy, and the so‐called “progeny or daughter” droplets are generated [16]. The size of nanoparticles is limited by the balance between surface energy (favors larger droplets) and electrostatic charge (favors smaller droplets) [17, 18]. Achieving ultra‐small sizes requires balancing competing forces while reducing instabilities, requiring precise control of the EHD jetting process.

Among various parameters that influence the size of nanoparticles, the lyotropic properties of the jetting solution play a significant role. Salinity affects the formation and size of nanoparticles during EHD jetting by enhancing the solution's conductivity, which impacts the jetting dynamics under an applied electric field. Molecular dynamics simulations explained the effect of NaCl on the size of Met‐enkephalin/water electrospray nanodroplets [19]. Similarly, an experimental study reported that collagen microparticles formed in the presence of CaCl_2_ were smaller than those formed with NaCl [20]. The effect of salinity on the structure and charge state of protein molecules has also been reported in similar processes like electrospray ionization (ESI) mass spectroscopy (MS) [21]. Yet, a systematic assessment of the role of salts in influencing the size and distribution of EHD jetted nanoparticles remains unexplored.

The Hofmeister Series gives an ordering of various anions and cations based on their ability to cause protein precipitation or aggregation. The typical ordering of anions follows the trend as: CO_3_^2−^ > SO_4_^2−^ > S_2_O_2_^−3^> H_2_PO_4_^−^ > F^−^ > Cl^−^ > Br^−^ ∼ NO_3_^−^ > I^−^ > ClO_4_^−^ > SCN^−^. The salts on the left end of the Hofmeister Series are kosmotropes that enhance or promote the degree of H‐bonding in water molecules and promote the formation of a hydration layer (water‐ion interactions are more favorable than ion‐protein interactions) and the salts on the right end are chaotropic, which tend to disturb the H‐bonding of water molecules [22, 23]. Herein, we aim to systematically investigate the influence of salinity and the Hofmeister Series on the size of human serum albumin (HSA) nanoparticles produced via EHD jetting.

## 2 Results and Discussion

### 2.1 Synthetic Protein Nanoparticles (SPNPs) via Electrohydrodynamic (EHD) Jetting

**FIGURE 1:**
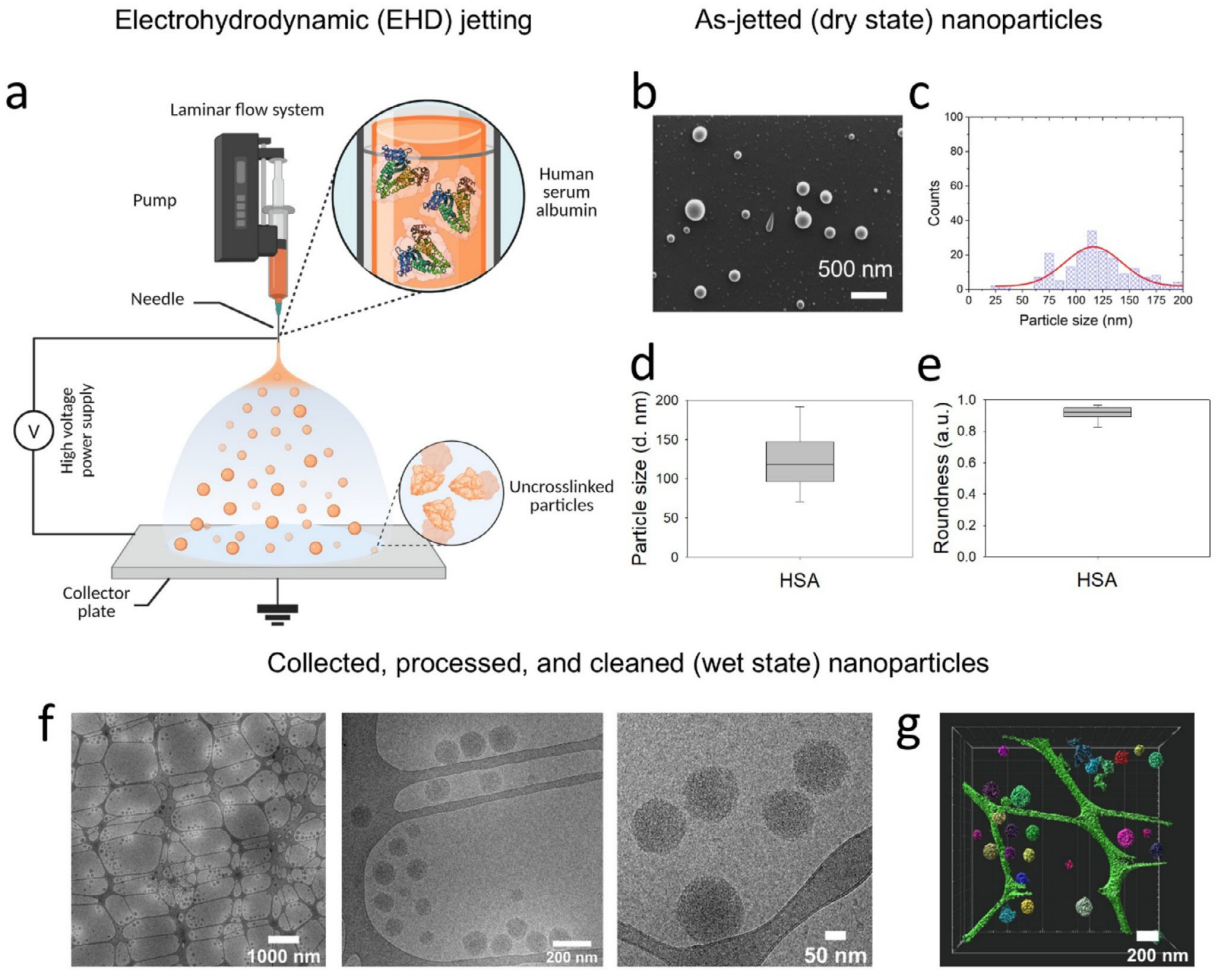
Synthesis and characterization of synthetic protein nanoparticles (SPNPs). (a) Schematic representation of the synthesis of human serum albumin (HSA) SPNPs using electrohydrodynamic (EHD) jetting. After jetting onto an aluminum collection pan, the particles were crosslinked via vapor‐phase glutaraldehyde (GA) treatment to produce water‐stable, crosslinked SPNPs. The schematic was created with BioRender.com. (b–e) Characterization of as‐jetted non‐crosslinked SPNPs showing (b) the morphology of the SPNPs using Scanning Electron Microscopy (SEM), scale: 500 nm, (c) the SPNPs size distribution curve, (d) particle diameters calculated from SEM images (n = 200), and (e) the morphology of SPNPs in terms of roundness. (f,g) Characterization of GA‐crosslinked SPNPs in the hydrated state showing (f) the Cryo‐transmission electron microscopy (cryo‐TEM) images of SPNPs at different magnifications, and (g) the 3D cryo‐electron tomogram of the SPNPs.

Human serum albumin (HSA) nanoparticle‐based drug delivery systems have observed significant developments in the past decade. HSA nanoparticles constitute a major segment of carriers in nanomedicine, thanks to their enhanced retention and permeability, and targeted drug and gene delivery. We prepared HSA‐based synthetic protein nanoparticles (SPNPs) using electrohydrodynamic (EHD) jetting (Figure 1a), as previously described [8, 9, 24]. Herein, a dilute solution of HSA (1% w/v) was prepared using a 20% v/v methanol aqueous co‐solvent system. Adding organic solvent to an aqueous protein solution has been shown to reduce the solution's surface tension and dielectric constant, making it suitable for EHD jetting [25]. EHD jetting of the HSA solution resulted in the deposition of solid SPNPs on the surface of the collection plate. Figure 1b–h shows the morphology and size‐related properties of the HSA SPNPs prepared using EHD jetting both in dry (as‐jetted, pre‐collection) and wet states (after‐collection, suspended in water). In the dry state, HSA SPNPs are relatively small (128 ± 47 nm average diameter calculated from the SEM images, number of particles = 200, shown in Figure 1b,d), polydisperse (broad size distribution curve shown in Figure 1c), and spherical (roundness > 0.9, Figure 1e). For collection, the SPNPs were placed in a sealed container for crosslinking using vapors of aqueous glutaraldehyde (20% v/v) for 1 h. After crosslinking, the SPNPs were collected from the aluminum plates and suspended in phosphate buffer saline (PBS) supplemented with 0.01% v/v Tween 20. SPNPs were then resuspended in ultrapure water using multiple ultracentrifugation and sonication steps.

Figure 1f,g shows typical 2D transmission electron microscopy (TEM) images and image‐processed 3D cryo‐electron (cryo‐ET) tomogram, respectively, of the SPNPs in the wet state. SPNPs are color‐coded based on their sizes. The TEM and cryo‐ET data confirm that the SPNPs are stable, non‐aggregating, and conform to the size reported in their dry state (120 ± 22 nm calculated from TEM images vs. 128 ± 47 nm calculated from SEM images). The insignificant difference between the dry and wet state diameters of the SPNPs is consistent with successful glutaraldehyde crosslinking. The real‐time cryo‐ET tomogram shows that the SPNPs are non‐agglomerating or stable in a 3D organization. It further highlights that the SPNPs prepared using EHD jetting do not have smooth curvatures, rather, they exhibit peripheral roughness.

### 2.2 Engineering the Size of SPNPs

#### 2.2.1 Increased Salinity Reduces the Size of SPNPs

**FIGURE 2:**
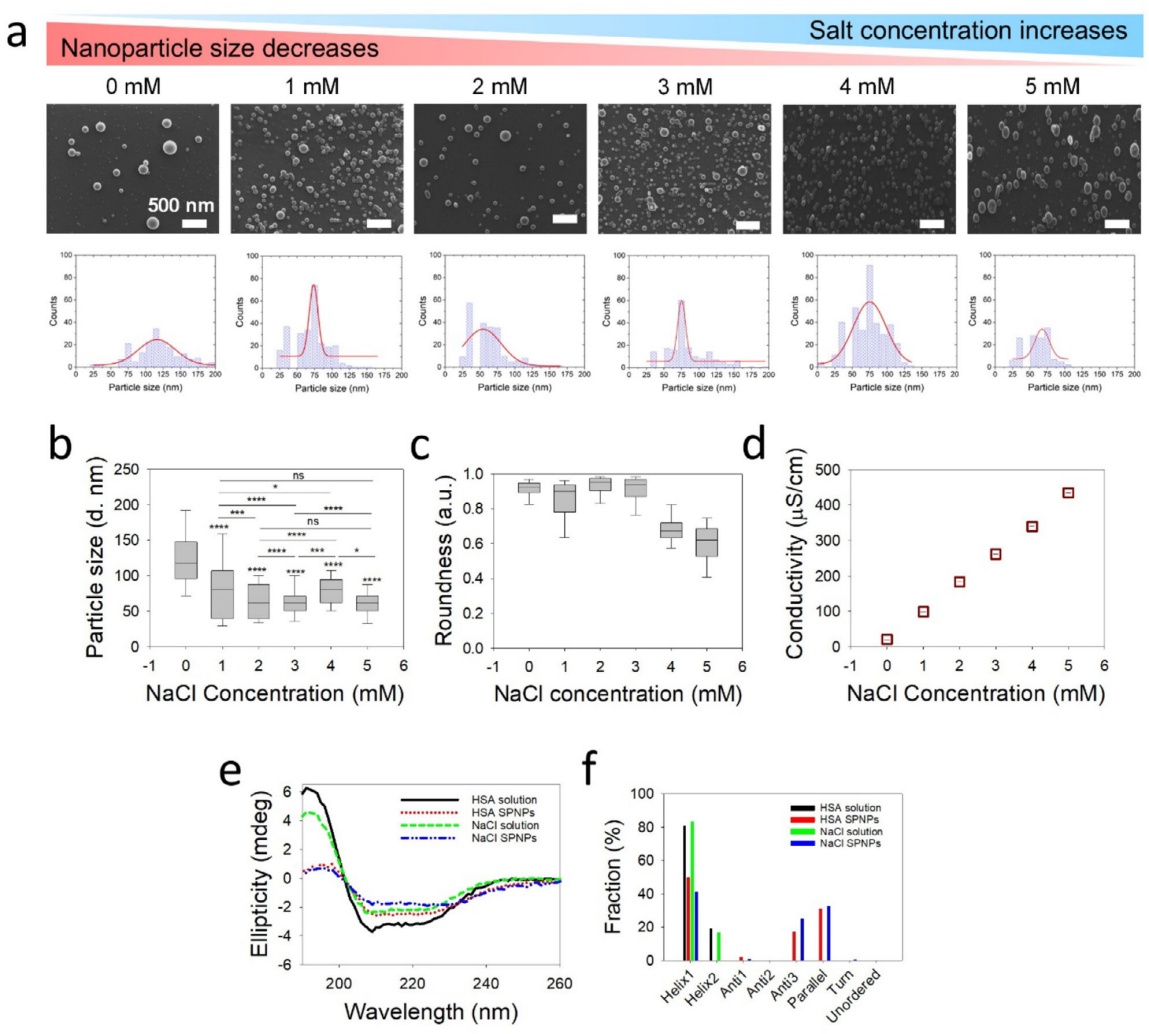
Higher salinity reduced the diameter of HSA synthetic protein nanoparticles (SPNPs). (a) Representative SEM images and the corresponding size distribution curves of as‐jetted, non‐crosslinked HSA SPNPs prepared from jetting solutions with varying concentrations of NaCl. Scale bar: 500 nm. (b) The mean diameters of as‐jetted SPNPs and (c) roundness values as a function of NaCl concentration, calculated from the respective SEM images (n = 200 SPNPs per condition). Statistical significance was assessed using Student's *t*‐test; comparisons to the no‐salt (0 mm) condition are indicated above each box, with additional comparisons shown using overhead lines. The significance levels, calculated using a Student's *t*‐test, are assigned as *p* ≤ 0.05 (^*^), *p* ≤ 0.01 (^**^), *p* ≤ 0.001 (^***^), *p* ≤ 0.0001 (^****^), and “ns” for not significant. (d) Electrical conductivity measurements of the HSA jetting solutions prepared with 1–5 mm NaCl in 20% v/v aqueous methanol. (e) Circular dichroism (CD) spectra of protein jetting solutions and the corresponding SPNPs suspended in ultrapure water. “HSA solution” refers to HSA dissolved in 20% v/v aqueous methanol, while “NaCl solution” refers to HSA dissolved in 20% v/v aqueous methanol containing 2 mm NaCl. “HSA SPNPs” and “NaCl SPNPs” represent nanoparticles prepared from the respective solutions. (f) Secondary structure fractions derived from the corresponding CD spectra, determined using BestSel analysis.

In this systematic study, we show that mixing small amounts of salt, i.e., NaCl in HSA jetting solutions, results in tight control over the size and size distribution of HSA SPNPs prepared via EHD jetting (Figure 2). Figure 2a shows the representative SEM images of the as‐jetted dry‐state SPNPs with different salt concentrations tested and their respective size distribution curves. Figure 2a,b shows that lower concentrations (1–3 mm) of NaCl reduce the size of as‐jetted nanoparticles and give narrower size distributions compared to the SPNPs prepared without salt (0 mm) or with higher salt concentrations (4–5 mm). In this case, a salt concentration of 3 mm NaCl resulted in the smallest SPNP sizes (61.2 ± 24.7 nm) and narrow size distributions. A jetting solution with 2 mm NaCl resulted in a similar size (60.4 ± 26.6 nm), but the distribution curve was comparatively broader (vs. 3 mm NaCl). The difference (2 mm vs. 3 mm NaCl) in nanoparticle population sizes appeared to be significant (*p*‐value < 0.0001, see Table S1). The addition of NaCl also influenced the shape of the SPNPs. The median roundness values were > 0.8 for NaCl ≤ 3 mm, i.e., the particle shape is close‐to‐perfect spherical (Figure 2c). However, for SPNPs prepared from jetting solutions containing 4–5 mm NaCl, the median roundness values were below 0.7, i.e., the SPNPs were less spherical in shape. Higher NaCl concentrations resulted in more irregular particle shapes (Figure 2a; Figure S1). For higher salt concentrations, it became increasingly difficult to achieve a stable jetting regime (Taylor cone), which can be attributed to decreased charge relaxation times at higher solution conductivity [26]. Figure 2d reveals the increase in electrical conductivities of HSA solutions by increasing NaCl concentrations.

We used circular dichroism (CD) spectroscopy to investigate potential changes in the secondary structure of HSA due to EHD jetting, collection, and processing of various SPNPs. The secondary structure of HSA, predominantly in the alpha‐helix form, exhibited characteristic optical activity in the far‐UV spectrum, with two negative bands at 208 and 222 nm, and a positive signal at 193 nm. Figure 2e shows the CD spectra of HSA SPNPs prepared from jetting solutions without salt (0 mm NaCl) and with salt (2 mm NaCl), referred to as HSA SPNPs and NaCl SPNPs, respectively, along with the spectra of the corresponding jetting solutions. When comparing the jetting solutions with the crosslinked SPNPs suspended in water, no significant changes in the positions of the negative bands at 208 and 222 nm were observed, except for a reduction in the intensity of the positive peak at 193 nm in the case of the SPNPs. To analyze these subtle differences, secondary structural fractions were extracted using BestSel (Figure 2f). A reduction in alpha‐helix content was noted when comparing jetting solutions to the corresponding SPNPs, which was attributed to the strong crosslinking of the SPNPs rather than the EHD jetting, collection, or processing steps [27, 28]. Notably, the presence of 2 mm NaCl in the jetting solution did not significantly affect the secondary structure of HSA (Figure 2e,f).

The relationship between the jetting solution's salinity and nanoparticle size is intricate, with multiple contributing factors to be considered. To gain a deeper understanding of why the presence of NaCl produces smaller SPNPs, we performed electrical conductivity measurements of the HSA jetting solutions. The addition of salt, i.e., 1–5 mm NaCl, in the HSA jetting solutions showed a linear increase of the electrical conductivity (Figure 2d). Indeed, higher electrical conductivity can result in smaller droplets due to a narrowing of the expelled fluid jet [26], which can be expected to contribute to decreased nanoparticle sizes in the presence of NaCl (Figure 2b). In the case of Coulombic fission of a single levitated microdroplet, the ions increased the surface conductivity of the parent droplet. For increasing ion concentrations, increased conductivity of the parent droplets, increased charge‐to‐mass ratios, and decreased sizes of resultant progeny droplets have been reported in literature [29, 30, 31]. Although electrical conductivity appears to be the most direct parameter influencing SPNP size, other physicochemical factors such as solution viscosity, surface tension, and evaporation rate can contribute to the jetting dynamics and droplet formation. To minimize confounding effects, all jetting solutions were prepared with a constant solvent composition (20% v/v methanol in water) and fixed protein concentration (1% w/v HSA).

**SCHEME 1:**
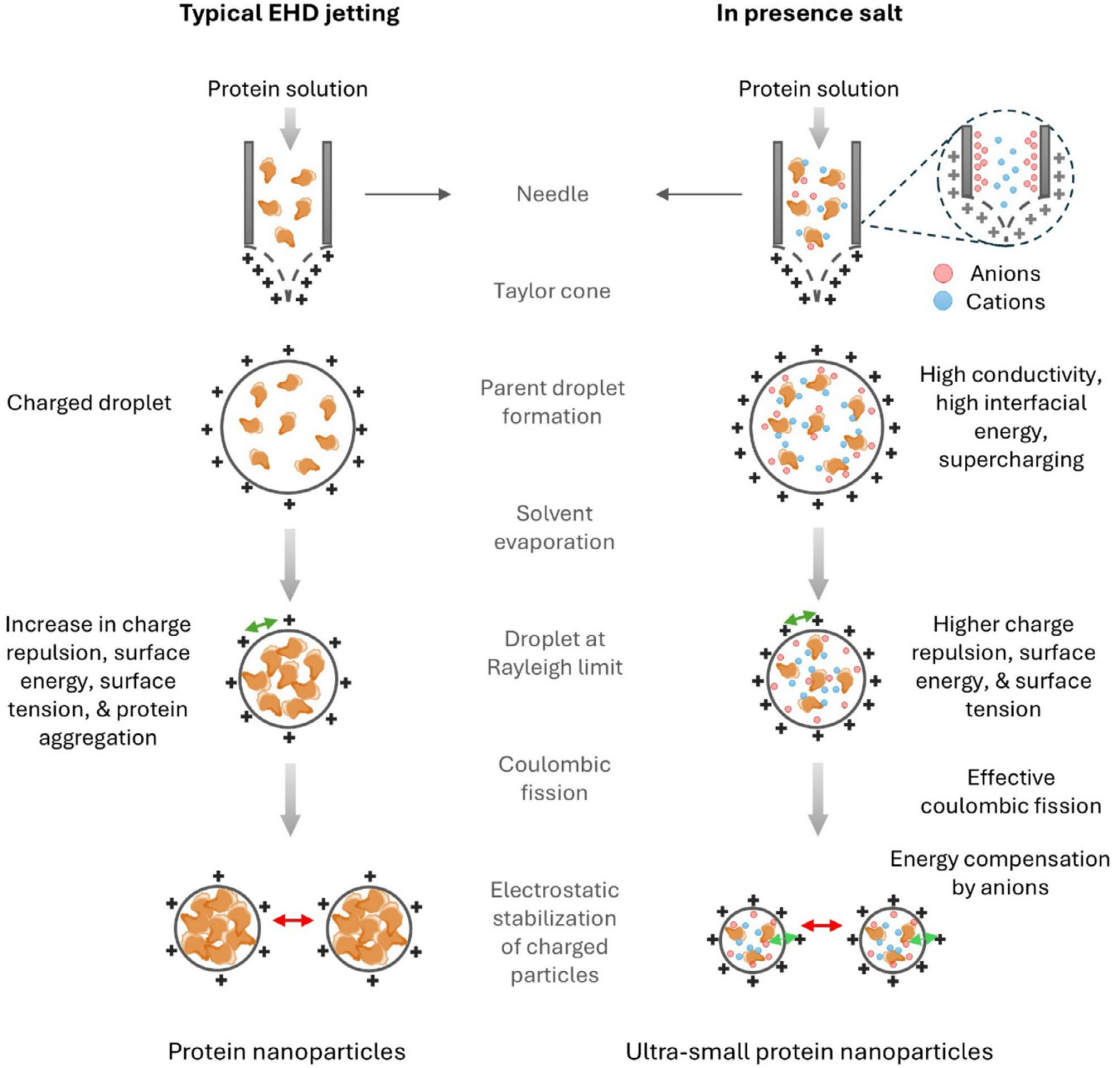
Schematic illustration of nanoparticle formation during electrohydrodynamic (EHD) jetting and the influence of salinity on particle size. Salinity enhances the conductivity of the jetting solution and interfacial charge density on the electrospray droplets. As the droplet size decreases, the surface energy and surface tension increase, which are energetically unfavorable phenomena. The salt ions compensate for the high surface energy required to achieve ultra‐small SPNPs.

Scheme 1 illustrates that during a typical EHD jetting process, i.e., without the addition of salt, there is a limit to how small nanoparticle sizes can be. Assuming a Taylor cone jetting mode, the jet breaks up into droplets, which are carried toward the grounded collector by the applied electrical potential, and the applied electrical charge is distributed among the droplets. Droplet size may decrease through two main mechanisms: Coulombic fission, which occurs when the charge on a droplet exceeds the Rayleigh limit [16], and solvent evaporation. Beyond the Rayleigh limit, the charge–charge repulsions overcome the surface tension, causing the droplet to split (fission event). However, further size reduction becomes difficult due to the increased overall surface energy of the droplets [17, 18].

In the presence of salt, charged ions play a pivotal role in stabilizing the jet, influencing the breakup mechanism, and ultimately determining the size of the resulting droplets or nanoparticles. High‐conductivity ions facilitate faster charge redistribution, reducing the charge relaxation time and improving jet stability, which leads to finer jet breakup and the formation of smaller, more uniform particles. Since the needle carries a positive polarity, high‐mobility anions accelerate toward the needle walls, increasing the interfacial charge density and electrostatic energy of the jetting solution [32]. Once droplets form, salt ions inside the droplets migrate to the liquid‐air interface, where they screen the surface charges. This reduces Coulombic repulsion and helps the system achieve a low‐energy, stable state, further promoting the formation of ultra‐small, more uniform SPNPs (Scheme 1).

However, salt ions may also influence protein–protein interactions during the EHD jetting process. As the droplets move toward the grounded collector, solvent evaporation causes them to shrink. During this phase, salt ions stabilize the charges on protein molecules [33], altering the balance of protein–protein interactions (Scheme 1). Although salting‐out typically causes protein precipitation at high salt concentrations, in this case, no significant protein–protein attractions or aggregation were observed in solution at low salt concentrations (1–5 mm). Positive values of the second virial coefficient, indicating the prevention of protein–protein attraction, have been reported for bovine serum albumin at low salt concentrations (0.125–0.25 m) over a broad pH range (5.5–8) [34, 35].

Similarly, other processes have reported the influence of salinity on the structure and charge state of proteins. In Electrospray Ionization (ESI), the presence of salt ions demonstrated an increase in electrical conductivity, leading to protein supercharging [21]. In Liquid Chromatography‐Mass Spectrometry (LC‐MS), supercharging reagents were added to the mobile phase to reduce the ion mass‐to‐charge (m/z) ratio (or equivalently, increase the charge‐to‐mass ratio) and improve detection efficiency [36]. Likewise, in ESI‐MS, highly charged and compact gaseous protein ions can be generated from aqueous buffered solutions containing salts, such as ammonium acetate or bicarbonate [37]. Among various supercharging reagents [38], salts can directly act as supercharging agents. Notably, studies have shown that adding small amounts (1–10 mm) of NaCl during ESI‐MS induces protein supercharging, which can lead to partial unfolding of the protein structure [21]. Transient changes in the secondary structure of HSA protein were observed when EHD jetting was performed directly onto quartz substrates, specifically at higher salt concentrations (Figure S2). In contrast, CD spectroscopy did not reveal significant alterations in the secondary protein structure for glutaraldehyde‐crosslinked SPNPs (Figure 2e,f), hinting toward a potentially protective effect of glutaraldehyde crosslinking. Similarly, HSA showed no significant structural changes when uncrosslinked SPNPs were redissolved in water (Figure S2). These findings offer important mechanistic insights into protein behavior during the EHD process, but may be difficult to generalize to other systems.

We also tested a range of flow rates (40, 60, 80, 100, and 120 µL h^−1^) for EHD jetting of the HSA jetting solutions with a fixed salt concentration of 3 mm NaCl (Figure S3). The size of the SPNPs remained consistently below 100 nm across all tested flow rates, but the narrowest size distributions were observed at flow rates of 60 and 100 µL h^−1^. Flow rate is a key parameter influencing particle size and distribution, with specific flow rates leading to narrower size distributions as previously reported [26]. Therefore, we fixed the flow rate at 60 µL h^−1^ throughout this study.

#### 2.2.2 Hofmeister Series Infers the Size of HSA SPNPs

Hofmeister anions may influence the size of human serum albumin (HSA) SPNPs during Electrohydrodynamic (EHD) jetting due to their effects on protein structure, solvent properties, and electrical conductivity of the jetting solution. Herein, we are interested in assessing the effect of Hofmeister salts on nanoparticle size.

**FIGURE 3:**
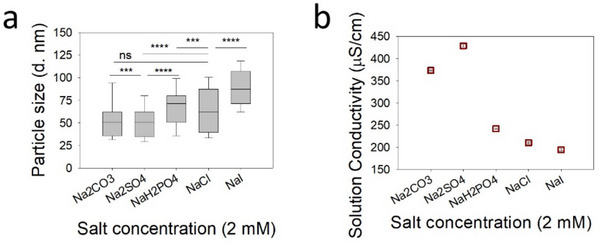
Hofmeister series (HS) anions modulated the size of HSA SPNPs. Evolution of mean SPNP diameters as a function of (a) anions with Na^+^ salts of the HS series at 2 mm concentration. (b) Electrical conductivity measurements of the HSA jetting solutions of various anions with Na^+^ salts of the HS series at 2 mm concentration, prepared in 20% (v/v) aqueous methanol.

We investigated the effect of five different Hofmeister anions, i.e., carbonate, sulfate, phosphate, chloride, and iodide, as shown in Figure 3a, on the size of the SPNPs, while keeping Na^+^ fixed as a cation. Figure S4 shows the evolution of average particle diameters with increasing concentrations of different Hofmeister anions. As expected, an approximately similar (to NaCl) trend of decreasing particle size was observed for increasing concentrations (and consequently the conductivity of the jetting solutions) of each anion. It aligns with the previously observed effect of salinity on the nanoparticle size. Interestingly, Figure 3a shows that at a fixed yet lower salt concentration, i.e., 2 mm, we observe an approximately increasing trend in particle size depending on the nature of the anion from salting‐out to salting‐in (left to right on the Hofmeister series). Ideally, the trend for the particle diameter should follow CO_3_^2−^ < SO_4_^2−^ < H_2_PO_4_^−^ < Cl^−^ < I^−^. However, we observed the particle diameter trend as SO_4_^2−^ < CO_3_^2−^ < H_2_PO_4_^−^ < Cl^−^ < I^−^. We thus conducted electrical conductivity measurements (SO_4_^2−^ > CO_3_^2−^ > H_2_PO_4_^−^ > Cl^−^ > I^−^), as shown in Figure 3b. The differences among the nanoparticle size populations were statistically significant with p‐values of *p* ≤ 0.001 or *p* ≤ 0.0001 for most cases (Figure S5 and Table S1). The particle diameters and corresponding SEM images with size distribution curves of the as‐jetted SPNPs are presented in Figures S5 and S7, respectively.

The observed trend (SO_4_^2−^ < CO_3_^2−^ < H_2_PO_4_− < Cl^−^ < I^−^) reflected the influence of anions on electrical conductivity and protein‐solvent interactions. Kosmotropic ions (e.g., SO_4_^2−^ and CO_3_^2−^) enhanced solution conductivity more effectively, resulting in finer jetting and smaller droplets. In contrast, chaotropic ions (e.g., I^−^) tended to destabilize protein structures and were less effective at increasing conductivity, leading to larger droplets and particles. The general trend of electrophoretic mobility among anions in the Hofmeister series was SO_4_^2−^ < F^−^ < Cl^−^ < Br^−^ < I^−^ < ClO_4_− [23].

**FIGURE 4:**
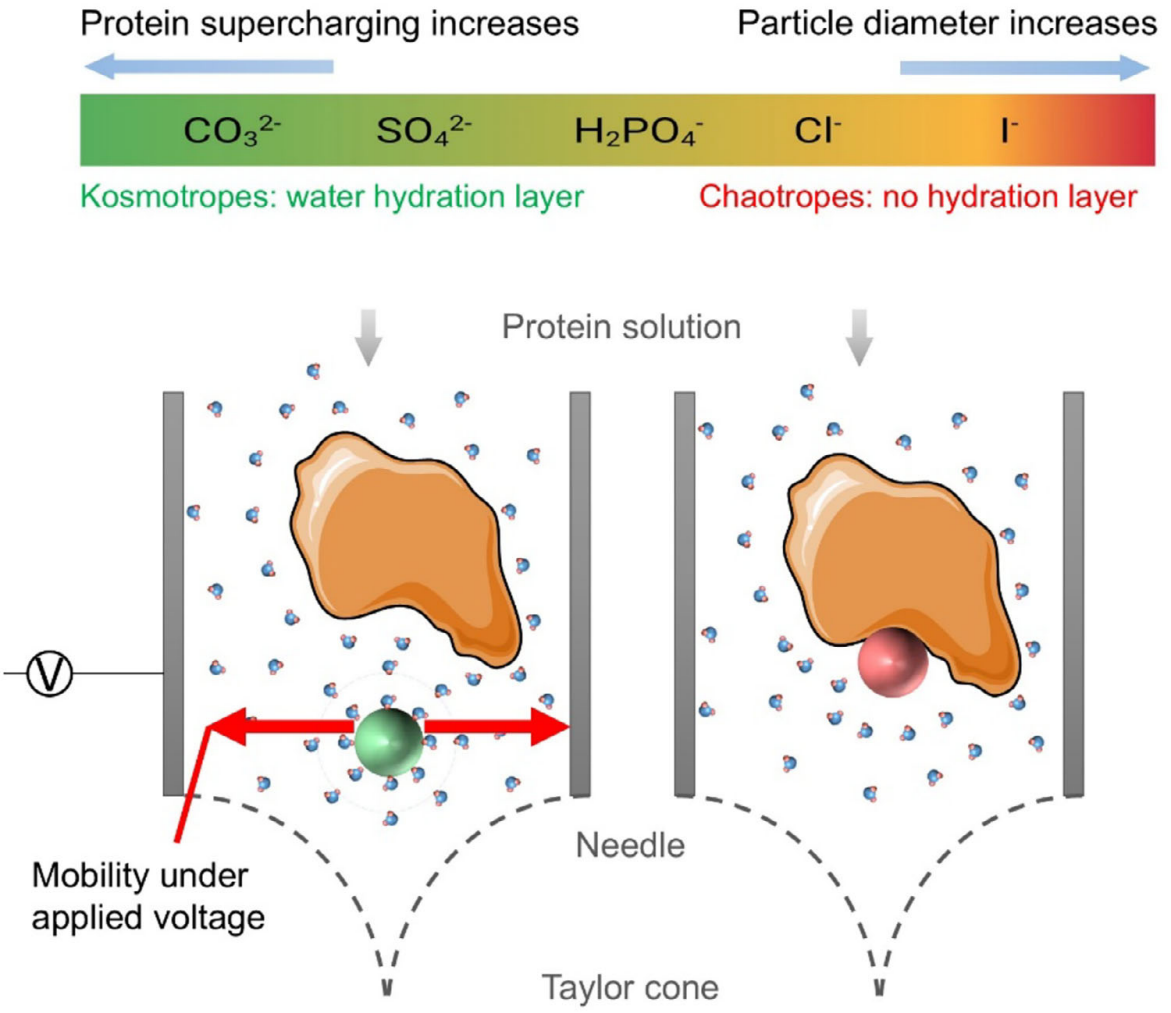
Hofmeister series of anions ordered according to their ability to form a water hydration layer, i.e., from right to left, contributed to protein supercharging and particle diameter. Protein‐ion and ion‐water interactions are shown during the EHD jetting process, where the needle is connected to a positive voltage source. The green sphere (left) and red sphere (right) represent kosmotropic and chaotropic anions, respectively.

As shown in Figure 4, strongly hydrated kosmotropic anions (e.g., SO_4_^2−^), which are known to form robust hydration layers [39], tend to be less likely to engage in direct association with proteins (salting‐out effect). Sulfate ions exhibit a stronger kosmotropic character, as evidenced by their higher surface tension increment values [40]. Concomitantly, sulfate ions tend to be repelled from the air/water interface due to their preferential interaction with water molecules. During EHD, this repulsion can contribute to the accumulation of ions near or at the interface, and charge–charge repulsion can influence surface tension. These effects are expected to contribute to more efficient jet thinning and droplet fission, thereby producing smaller nanoparticles. Carbonate, while also kosmotropic, is a stronger base than sulfate and might be less efficient in promoting such interfacial effects. Additionally, electrostatic interactions between carbonate ions and the negatively charged HSA surface could reduce interfacial mobility and contribute to slightly larger particles. These differences in interfacial behavior and protein‐binding affinity likely contribute to the observed reversal in nanoparticle size trend between sulfate and carbonate. Conversely, weakly hydrated chaotropes (e.g., I^−^) disrupt structured water molecules and preferentially associate with protein molecules (salting‐in effect). These salts resulted in larger SPNPs.

The influence of Hofmeister anions on the supercharging of proteins during electrospray ionization is an important consideration when understanding SPNP sizes. Studies investigating the effect of different Hofmeister anions on electrothermal protein supercharging from aqueous sodium or ammonium salt solutions revealed a reverse Hofmeister trend: sulfate > hydrogen phosphate > thiocyanate > bicarbonate > chloride > bromide > acetate > iodide > perchlorate [37]. At a given concentration, anions like sulfate, carbonate, and phosphate, which have a higher propensity for supercharging proteins, were found to produce smaller SPNPs. CD spectra and corresponding BestSel secondary structure fractions for all anion jetting solutions are shown in Figure S9, comparing the SPNPs to their respective free HSA jetting solutions. The CD spectra for anion‐containing solutions revealed similar changes compared to control HSA (without salt). Minor changes in the position and intensity of bands at 222, 208, and 193 nm were attributed to the chemical crosslinking of the SPNPs [27]. Figure S10 shows the effect of anions on the secondary structure of HSA during the EHD jetting process. On quartz substrates, the drop‐cast samples of free HSA jetting solutions with various anions displayed CD spectra resembling those of HSA without salt. However, EHD jetting of HSA solutions directly onto quartz surfaces resulted in drastic shifts in the characteristic bands of HSA, but only for jetting solutions containing different anions. Interestingly, once the crosslinked SPNPs were suspended in water, the CD spectra showed no significant changes compared to the corresponding jetting solutions. This finding highlights the protective effects of SPNPs on the secondary structure of HSA.

Cations exhibited a less pronounced effect on the size of the SPNPs. We investigated the impact of three different Hofmeister cations, i.e., ammonium, potassium, and magnesium on the size of SPNPs while keeping Cl^−^ fixed as the anion. As shown in Figure S4d, the diameters of the SPNPs remained consistently below 50 nm for all salt concentrations. The as‐jetted SPNPs displayed narrow size distributions, as calculated from the diameters (Figure S6) derived from SEM images (Figure S8). While electrical conductivity increased with all salt concentrations (Figure S4e), no systematic correlation with particle size was observed [41].

#### 2.2.3 Salt Does not Dictate the Shape of the SPNPs

To investigate whether the SPNPs produced from protein formulations with salts entrap the salts inside their structures, we performed a control experiment using ammonium bicarbonate (NH_4_HCO_3_). The resulting SPNPs were similar in size and shape to those produced under other salt conditions tested in this work. However, as the salt concentration increased from 1 to 3 mm, the mean nanoparticle diameter decreased, and the size distributions narrowed (Figure S11). No significant impact on the secondary structure of HSA was observed between the jetting solution and the SPNPs (Figure S12).

## 3 Conclusions

The systematic engineering of protein nanoparticle size and distribution is crucial for the use of protein nanoparticles in nanomedicine. In this study, we developed HSA SPNPs via EHD jetting and thoroughly investigated the influence of salinity on nanoparticle size and distribution. Our results demonstrated that a slight increase in salinity, achieved by adding 1–5 mm NaCl to the HSA jetting solution, produced SPNPs with narrower size distributions and particle diameters that were consistently below 100 nm. Additionally, we explored the lyotropic effects of various Hofmeister anions on nanoparticle size, highlighting the contrasting roles of kosmotropic and chaotropic anions. For kosmotropic anions, we propose that ion‐water interactions dominate, allowing these anions to migrate more effectively toward the liquid/air interface during jetting. The increased mobility of ions, rather than proteins, which are more hydrophobic and lower the surface tension of the nanodroplets during EHD jetting, promotes the formation of smaller SPNPs. While this study focuses on HSA, a protein known for its stability, negative charge at physiological pH, and high solubility, electrohydrodynamic jetting has been applied to a wide range of different proteins, including insulin, hemoglobin, or transferrin [24, 43]. Future studies involving proteins with diverse structural features, surface charges, and hydrophobicity profiles will be essential to inform the broader applicability of the herein observed trends.

## 4 Experimental Section

### 4.1 Materials

Albumin from human serum (HSA, lyophilized powder, essentially globulin free, ≥ 99%, product no. A8763) was purchased from Sigma. All buffers, purchased in solution form, and all other reagents/salts used were of lab grade and acquired from Sigma–Aldrich or Thermo Fisher.

### 4.2 Protein Jetting Solutions

Various jetting solutions of human serum albumin (HSA) at a final concentration of 10 mg mL^−1^, dissolved in methanol 20% v/v aqueous solution with (or without) small concentrations (1, 2, 3, 4, and 5 mm) of 9 different salts, (ammonium chloride (NH_4_Cl), potassium chloride (KCl), magnesium chloride (MgCl_2_), sodium chloride (NaCl), sodium iodide (NaI), monosodium phosphate (NaH_2_PO_4_), sodium carbonate (Na_2_CO_3_), sodium sulfate (Na_2_SO_4_), and ammonium carbonate (NH_4_HCO_3_)), were prepared.

### 4.3 Electrical Conductivity of Protein Jetting Solutions

Electrical conductivity measurements were performed on freshly prepared HSA jetting solutions with previously described 9 different salts with concentrations ranging from 1 to 5 mm. Conductivity was determined using a ThermoFisher Orion VersaStar Pro pH/Conductivity meter and a low‐volume conductivity probe from Microelectrodes, Inc. (Bedford, NH). Instrument calibration was verified using conductivity standards of 100 and 1000 µS cm^−1^. All measurements were independently repeated three times, and error bars represent standard deviation.

### 4.4 Electrohydrodynamic (EHD) Jetting

Human serum albumin (HSA) protein nanoparticles were synthesized using EHD jetting, as previously described [3, 25, 42, 43]. To produce nanoparticles, the protein solution at a final concentration of 10 mg mL^−1^ was pumped through four syringes (25G blunt tip capillary system) at a flow rate of 0.06 mL h^−1^, and a sufficient bias (voltage ranging approximately from 8.6 to 9.2 kV) applied between the needle and a grounded aluminum collecting plate. The applied voltage was set to a value suitable to achieve a stable Taylor cone. The voltage causes the meniscus to be pulled toward the collecting substrate (aluminum pans or stainless‐steel plates), and the stream subsequently breaks up into nanometer‐sized droplets via Coulombic fission. In mid‐flight, the solvents rapidly evaporate to form solid nanoparticles. The distance from the needle tip to the collection surface was 5 cm. The aluminum collection pans were then placed into a sealed container with 2.5 mL of glutaraldehyde (GA) 20% v/v aqueous solution for 1 h to achieve vapor‐phase crosslinking. For collection, the crosslinked nanoparticles from the aluminum pans were carefully scraped off using a solution of DPBS 1X supplemented with 0.01% v/v Tween 20. The collected particles were purified by ultracentrifugation and sonication steps. All experiments were independently repeated three times to ensure reproducibility.

### 4.5 Scanning Electron Microscopy (SEM)

Scanning electron microscopy was used to characterize the morphology of as‐jetted nanoparticles in their dry state, before collection and purification. To prepare samples for imaging, silicon wafers were placed atop the collection plate during EHD jetting for a suitable duration (from 5 to 30 min). The wafers were then placed on a copper‐covered scanning electron microscopy (SEM) stub, sputter coated with a layer of platinum (3 nm thickness), and then imaged using the TESCAN Vega 3 with a tungsten filament electron source. Images were taken using a working distance of 6 mm and 20 kV emission voltage. The resulting images were analyzed using ImageJ according to previously described methods [25]. Individual nanoparticles (n = 200) were analyzed to obtain the calculated morphological properties (roundness, diameter, and size distributions). All measurements were independently repeated three times to ensure reproducibility.

### 4.6 Cryo‐ Transmission Electron Microscopy (TEM) and Electron Tomography (ET)

Cryogenic Transmission Electron Microscopy work flow was performed similar to the previous work [44]. A home‐made plunge freeze apparatus was used to prepare grids for cryoTEM and cryoET. Typically onto 200‐mesh lacey carbon grids, 400 µL of nanoparticle solution was deposited. The excess liquid was removed by means of a Whatman filter paper applied to the front of the liquid drop. The grid was immediately plunged into liquid ethane, and then transferred to a Gatan 626 (Warrendale, PA, USA) gryogenic holder at −170°C. Imaging at −170°C was carried out in a Tecnai F20 microscope (Thermo Fisher, Hillboro, USA) operating at 200 kV and equipped with a Falcon III camera (Thermo Fisher, Hillsboro, USA). The tilt‐series acquired for reconstructing a 3D image (i.e. tomogram) of the sample was collected automatically from −60° to 60° with 2° angular increments at 19 000 × (camera's pixel size = 0.52 nm) using the defocus ∼ −3 µm and a total dose of ∼ 40 e−/Å [2]. They were drift corrected by the software of acquisition Tomo 4.0 (Thermo Fisher, Hillsboro, USA). Alignment and reconstruction were done with Inspect 3D (Thermo Fisher). The analysis of 3D images was performed with Imaris V.10.2 (Bitplane).

### 4.7 Circular Dichroism (CD) Analysis

The CD spectra measurements were performed mainly to analyze the secondary structure of proteins in free solutions (formulations prepared in methanol 20% v/v) and in crosslinked nanoparticles (clean SPNPs suspended in water). The samples were diluted to the required concentrations (10 µg mL^−1^) and measured in a 0.1 cm pathlength Hellma quartz cuvette in a Jasco J‐1500 CD Spectrometer. Spectra were acquired at a temperature of 20°C from 190 to 260 nm, using a data pitch of 0.2 nm, a bandwidth of 1 nm, and a scan speed of 20 nm/min. Each sample was measured for a total of 3 accumulations and normalized to the respective media. The average signals were analyzed for secondary structure using BestSel. Moreover, to investigate the effect of EHD jetting on the protein structure, CD spectra were recorded on samples prepared by directly electrospraying protein formulations on quartz substrates. Here, respective control samples were prepared by drop casting protein formulations (50 µg mL^−1^, 50 µL volume) on quartz substrates for 1 h and air dried. All measurements were repeated three times to ensure reproducibility.

## Conflicts of Interest

The authors declare no conflicts of interest.

## Supporting information




**Supporting File 1**: marc70020‐sup‐0001‐SuppMat.docx

## Data Availability

The data that support the findings of this study are available from the corresponding author upon reasonable request.
