# Supplementary material for: Lyotropy as a Design Consideration for Ultra‐Small Protein Nanoparticles via Electrohydrodynamic Jetting
Source: Macromol Rapid Commun. 2025 Aug 14;46(23):e00533. doi: 10.1002/marc.202500533 (PMC12687699; doi:10.1002/marc.202500533)
Supplement: Supplementary file 1 — Supporting File 1: marc70020‐sup‐0001‐SuppMat.docx [file MARC-46-e00533-s001.docx]

Supporting Information

Lyotropy as a Design Consideration for Ultra-small Protein Nanoparticles via Electrohydrodynamic Jetting

Muhammad Haseeb Iqbal,^a^ Julio Zelaya,^b^ Quy Ong,^c^ Francesco Stellacci,^c, d*^ and Joerg Lahann^a, b*^

^a^ M. H. Iqbal, J. Lahann

Institute of Functional Interfaces, Karlsruhe Institute of Technology, Eggenstein-Leopoldshafen, Germany, E-mail: [lahann@umich.edu](mailto:lahann@umich.edu)

^b^ J. Zelaya, J. Lahann
Biointerfaces Institute, University of Michigan, Ann Arbor, Michigan, United States

^c^ Q. Ong, F. Stellacci

Institute of Materials, Ecole Polytechnique Fédérale de Lausanne (EPFL), Lausanne, Switzerland

^d^ F. Stellacci

Bioengineering Institute, Ecole Polytechnique Fédérale de Lausanne (EPFL), Lausanne, Switzerland, E-mail: francesco.stellacci@epfl.ch


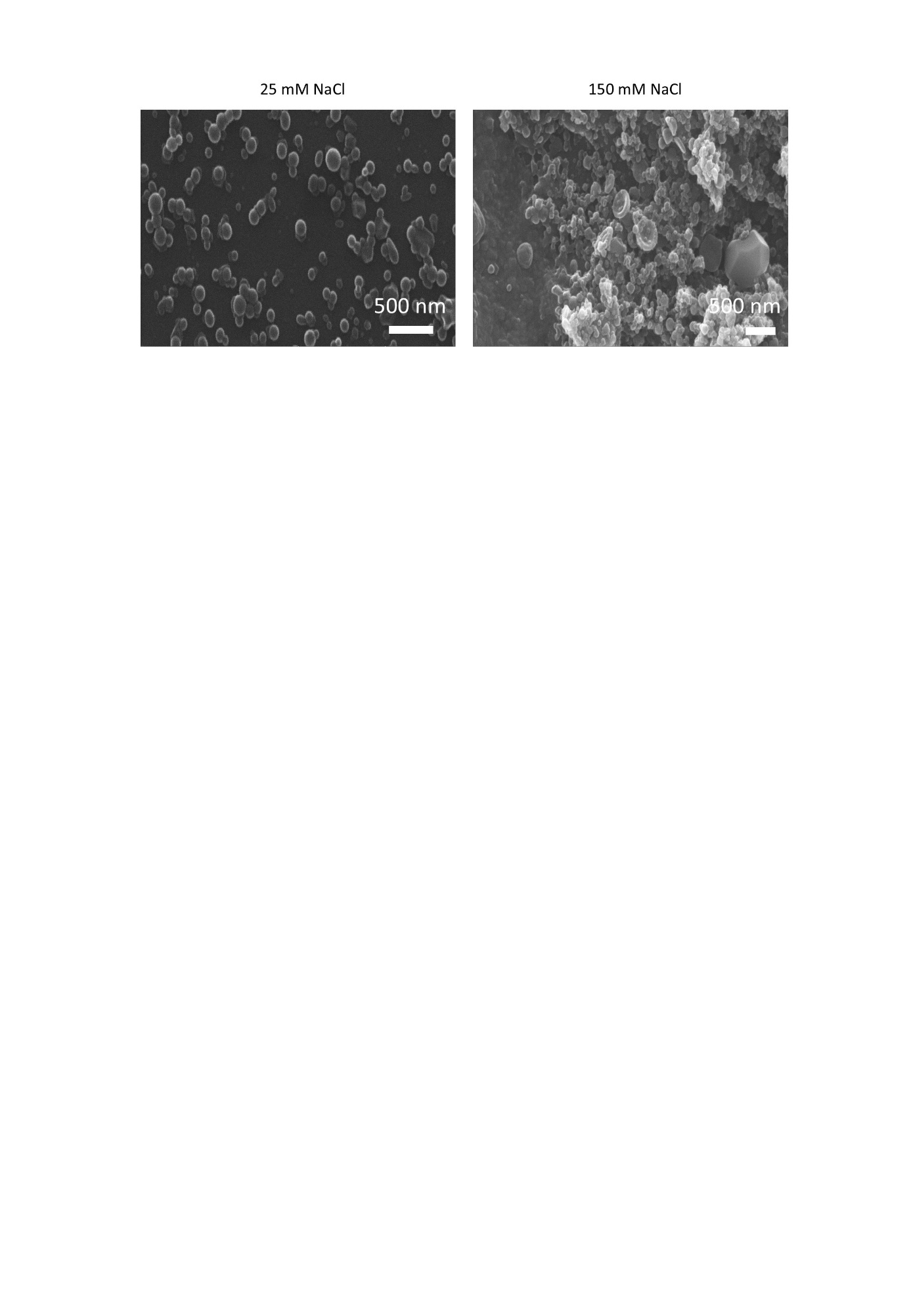


**Figure S1:** Scanning electron microscopy (SEM) images of as-jetted, non-crosslinked human serum albumin (HSA) synthetic protein nanoparticles (SPNPs) fabricated using jetting solutions containing different concentrations of NaCl in 20% v/v aqueous methanol. Scale bar: 500 nm.


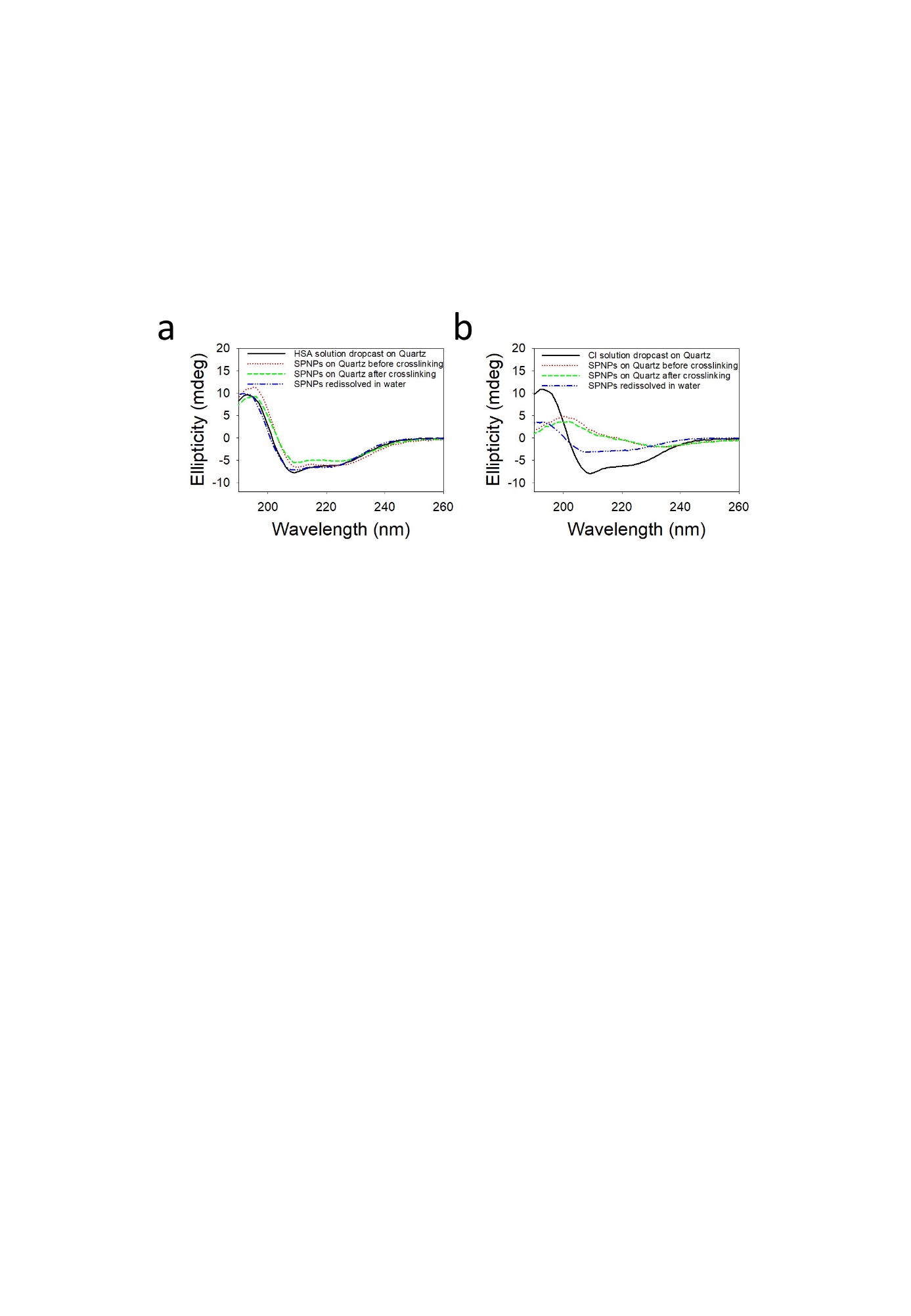


**Figure S2: Circular dichroism (CD) analysis of protein solutions and as-jetted SPNPs subjected to electrohydrodynamic (EHD) jetting, with and without NaCl.** CD spectra are shown for synthetic protein nanoparticles (SPNPs) prepared from (a) HSA without salt and (b) HSA solutions containing 2 mM NaCl in 20% v/v aqueous methanol. Samples include: protein jetting solutions (50 µL volume, 50 µg mL⁻¹) drop-cast and air-dried on quartz substrates; dry-state, uncrosslinked SPNPs deposited via EHD jetting onto quartz; dry-state, crosslinked SPNPs jetted onto quartz; and uncrosslinked SPNPs redissolved in ultrapure water.


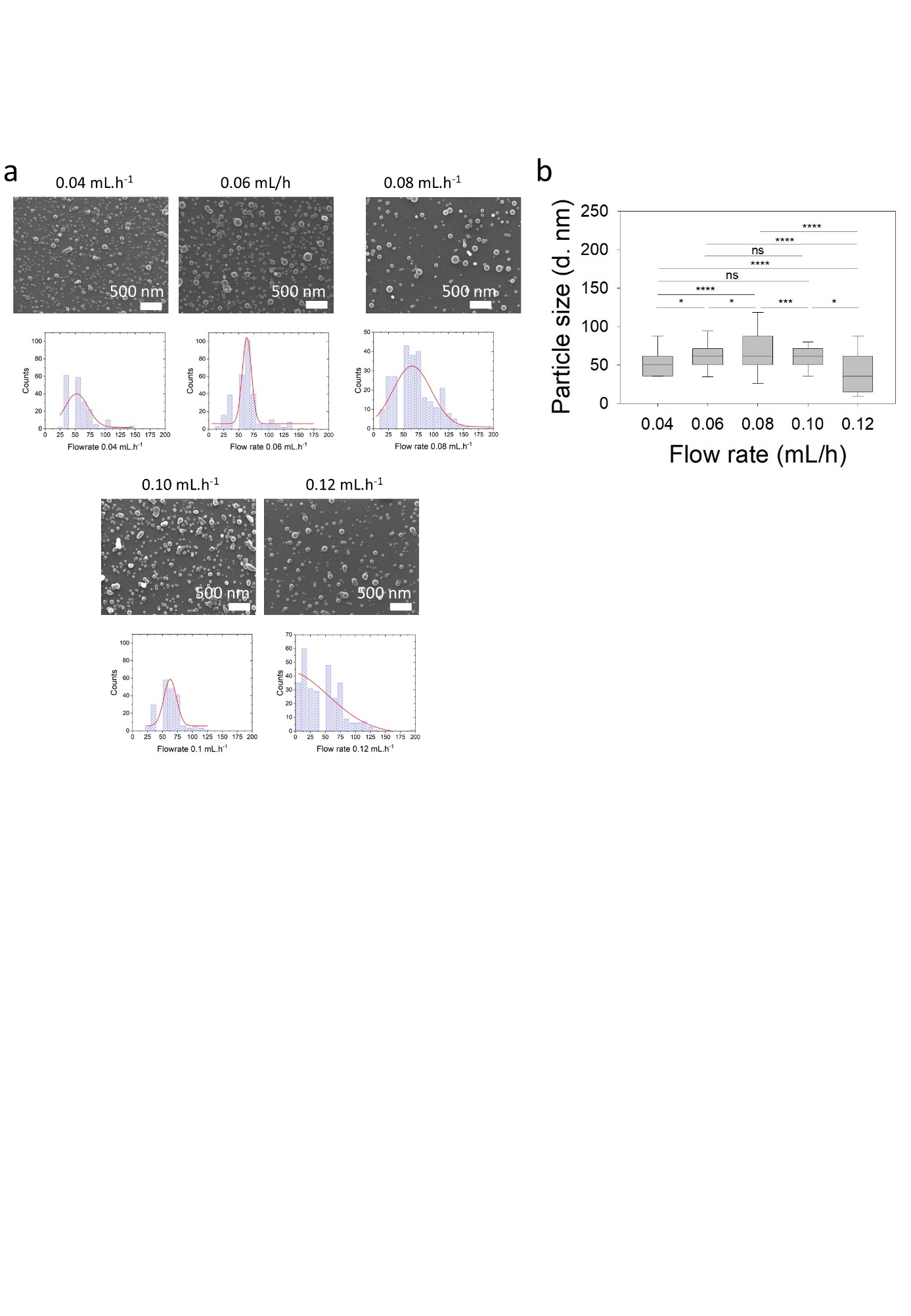


**Figure S3:** **Effect of flow rate on the diameters and size distributions of human serum albumin (HSA) synthetic protein nanoparticles (SPNPs).** (a) SEM images and corresponding size distribution histograms are shown for as-jetted, non-crosslinked SPNPs prepared from jetting solutions containing 3 mM NaCl. Scale bar: 500 nm. (b) The plot of average SPNP diameter versus applied flow rate is presented, with particle sizes determined from SEM images (n = 200 nanoparticles per condition). The significance levels, calculated using Student’s t-test, are assigned as p ≤ 0.05 (*), p ≤ 0.01 (**), p ≤ 0.001 (***), p ≤ 0.0001 (****), and “ns” for not significant.


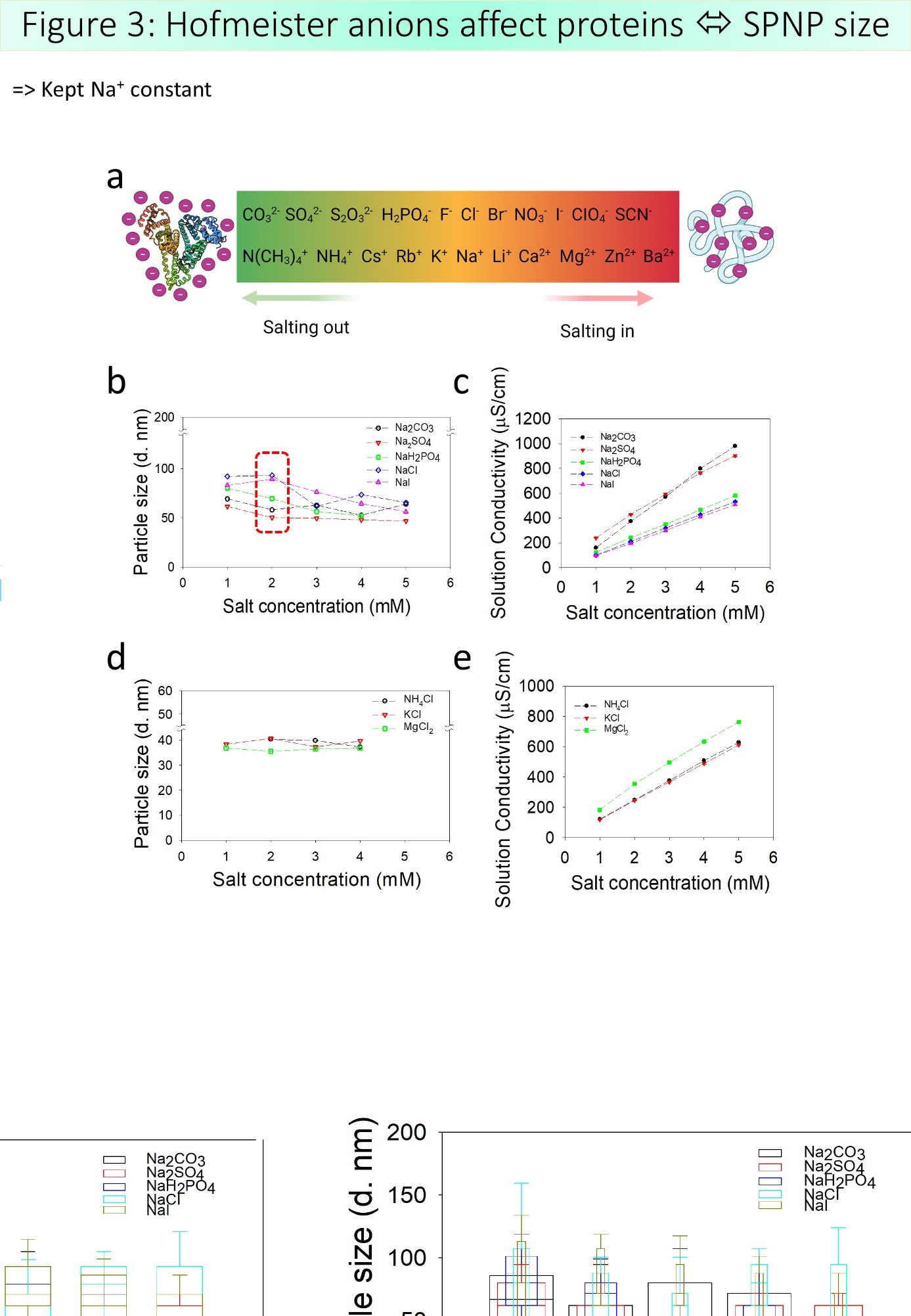


**Figure S4:** **Hofmeister series (HS) salts modulate the size of HSA synthetic protein nanoparticles (SPNPs).** (a) Schematic representation of the Hofmeister Series, illustrating the ordering of anions and cations based on their tendency to promote protein precipitation (salt-out) or solubilization (salt-in). (b, d) Plots showing the evolution of mean SPNP diameters as a function of increasing salt concentration (1–5 mM) for various (b) anions of Na⁺ salts and (d) cations of Cl⁻ salts corresponding to the HS. (c, e) Electrical conductivity measurements of HSA jetting solutions containing increasing concentrations (1–5 mM) of the corresponding (c) anions and (e) cations, prepared in 20% v/v aqueous methanol.


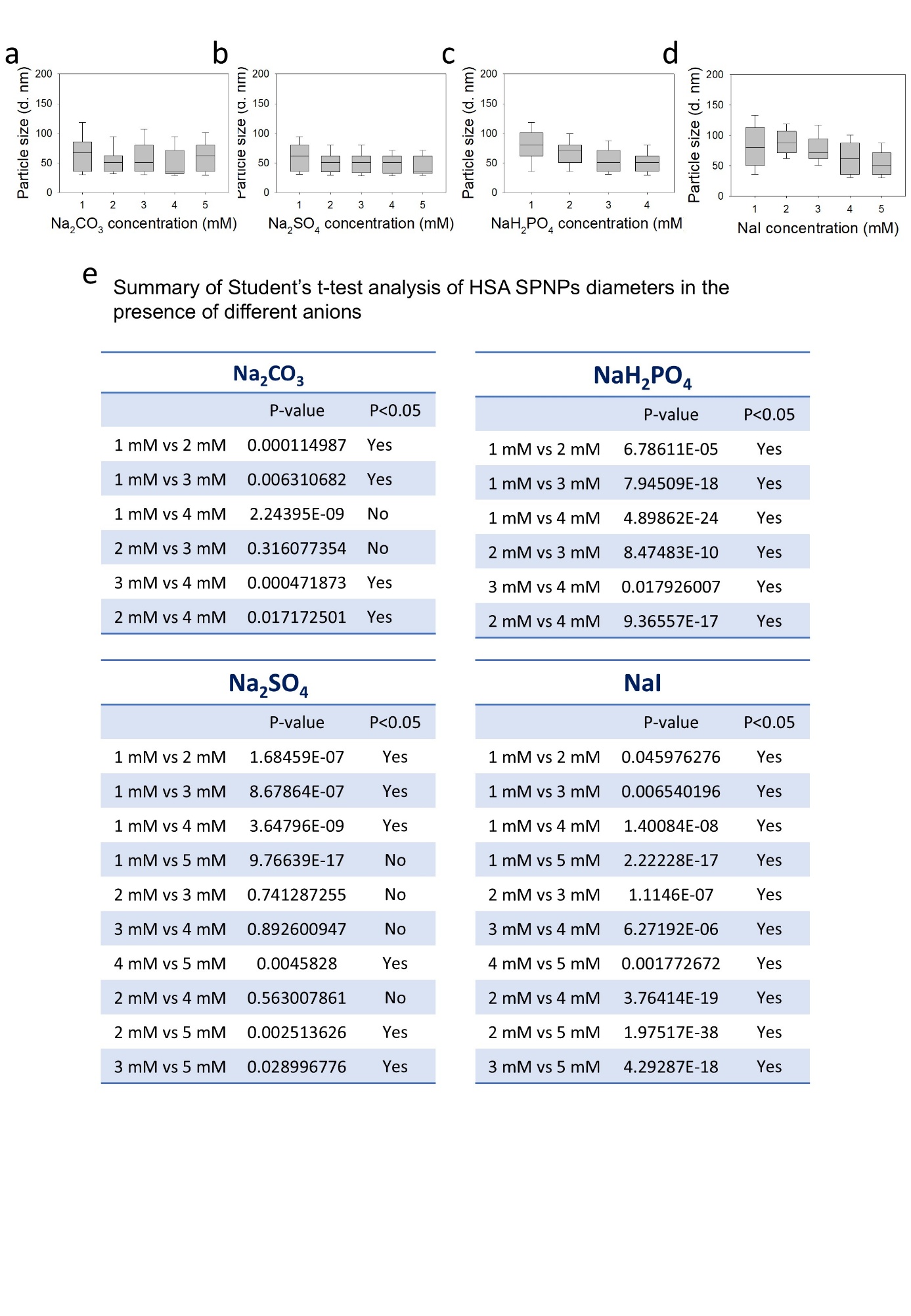


**Figure S5: Hofmeister anions modulate the size of HSA synthetic protein nanoparticles (SPNPs).** Evolution of mean SPNP diameters as a function of increasing salt concentrations (1–5 mM) for solutions, prepared in 20% v/v aqueous methanol, containing selected Hofmeister series anions: (a) Na_2_CO_3_, (b) Na_2_SO_4_, (c) NaH_2_PO_4_, and (d) NaI. Diameters were determined from scanning electron microscopy (SEM) images, with measurements taken from n = 200 nanoparticles per condition. (e) Summary of Student’s t-Test analysis of HSA SPNPs diameters for various Hofmeister anions.

**Table S1:** Summary of Student’s t-Test analysis of HSA SPNPs diameters in the presence of Hofmeister anions.

| **P-values** | | | | | |
| --- | --- | --- | --- | --- | --- |
|  | **Concentrations** | | | | |
|  | **1 mM** | **2 mM** | **3 mM** | **4 mM** | **5 mM** |
| Na_2_CO_3_ vs Na_2_SO_4_ | 0.004688459 | 0.000782223 | 3.20766E-09 | 0.013007717 | - |
| Na_2_CO_3_ vs NaH_2_PO_4_ | 0.000432706 | 3.06804E-06 | 0.07540101 | > 0.05 | - |
| Na_2_CO_3_ vs NaCl | > 0.05 | > 0.05 | 4.30602E-15 | 5.52139E-21 | - |
| Na_2_CO_3_ vs NaI | 3.72636E-05 | 1.81295E-29 | 3.28403E-10 | 6.93319E-06 | - |
| Na_2_SO_4_ vs NaH_2_PO_4_ | 3.43533E-11 | 3.84074E-18 | 0.002741484 | 0.002943877 | - |
| Na_2_SO_4_ vs NaCl | 0.002443664 | 1.14732E-05 | 3.84055E-24 | 8.38185E-51 | 1.66704E-07 |
| Na_2_SO_4_ vs NaI | 4.75116E-12 | 8.42697E-52 | 9.93251E-29 | 3.26194E-13 | 1.82147E-09 |
| NaH_2_PO_4_ vs NaCl | 1.49816E-05 | 0.000135605 | 2.03232E-22 | 8.42547E-33 | - |
| NaH_2_PO_4_ vs NaI | > 0.05 | 2.10777E-16 | 4.84879E-15 | 8.33243E-08 | - |
| NaI vs NaCl | 1.14537E-06 | 1.22568E-26 | 0.011242191 | 0.000151985 | 0.008180898 |
|  |  |  |  |  |  |
|  | **NaCl** | | | | |
| 1 mM vs 0 mM | 7.54879E-43 |  | 0 mM vs 5 mM | 6.17771E-33 |  |
| 1 mM vs 2 mM | 0.00089697 |  | 2 mM vs 3 mM | 6.02168E-15 |  |
| 1 mM vs 3 mM | 3.67243E-08 |  | 3 mM vs 4 mM | 0.000135575 |  |
| 1 mM vs 4 mM | 0.011409118 |  | 4 mM vs 5 mM | 0.02517791 |  |
| 1 mM vs 5 mM | 0.429164892 |  | 2 mM vs 4 mM | 3.69906E-11 |  |
| 0 mM vs 2 mM | 6.82268E-50 |  | 2 mM vs 5 mM | 0.178322957 |  |
| 0 mM vs 3 mM | 2.6059E-25 |  | 3 mM vs 5 mM | 1.0182E-05 |  |
| 0 mM vs 4 mM | 2.80283E-38 |  |  |  |  |
|  |  |  |  |  |  |


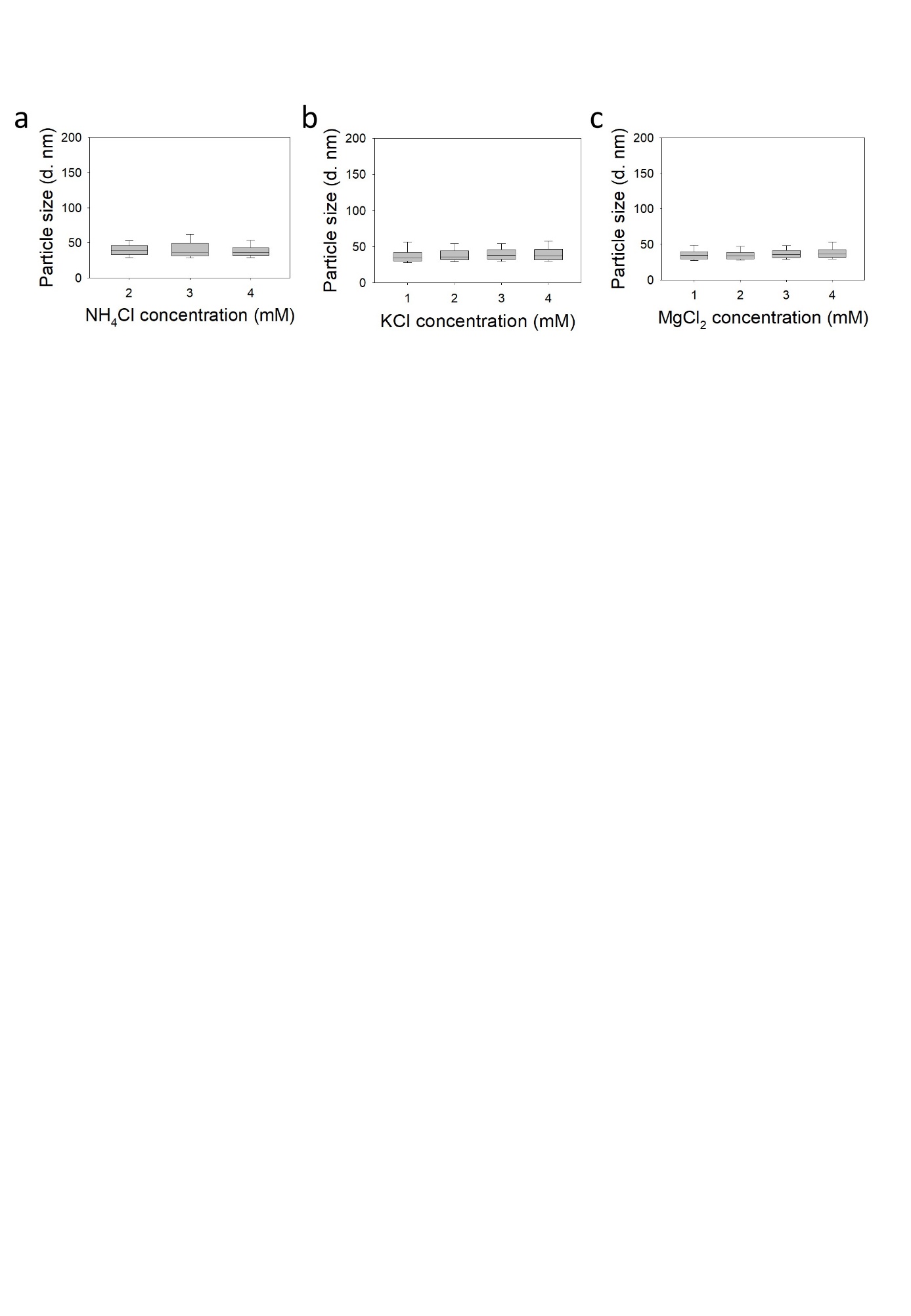


**Figure S6: Hofmeister cations modulate the size of HSA synthetic protein nanoparticles (SPNPs).** Evolution of mean SPNP diameters as a function of increasing salt concentrations (1–5 mM) for solutions containing selected Hofmeister series cations: (a) NH₄Cl, (b) KCl, and (c) MgCl₂. SPNPs were prepared using 20% v/v aqueous methanol as the solvent system. Diameters were determined from scanning electron microscopy (SEM) images, from n = 200 nanoparticles per condition.


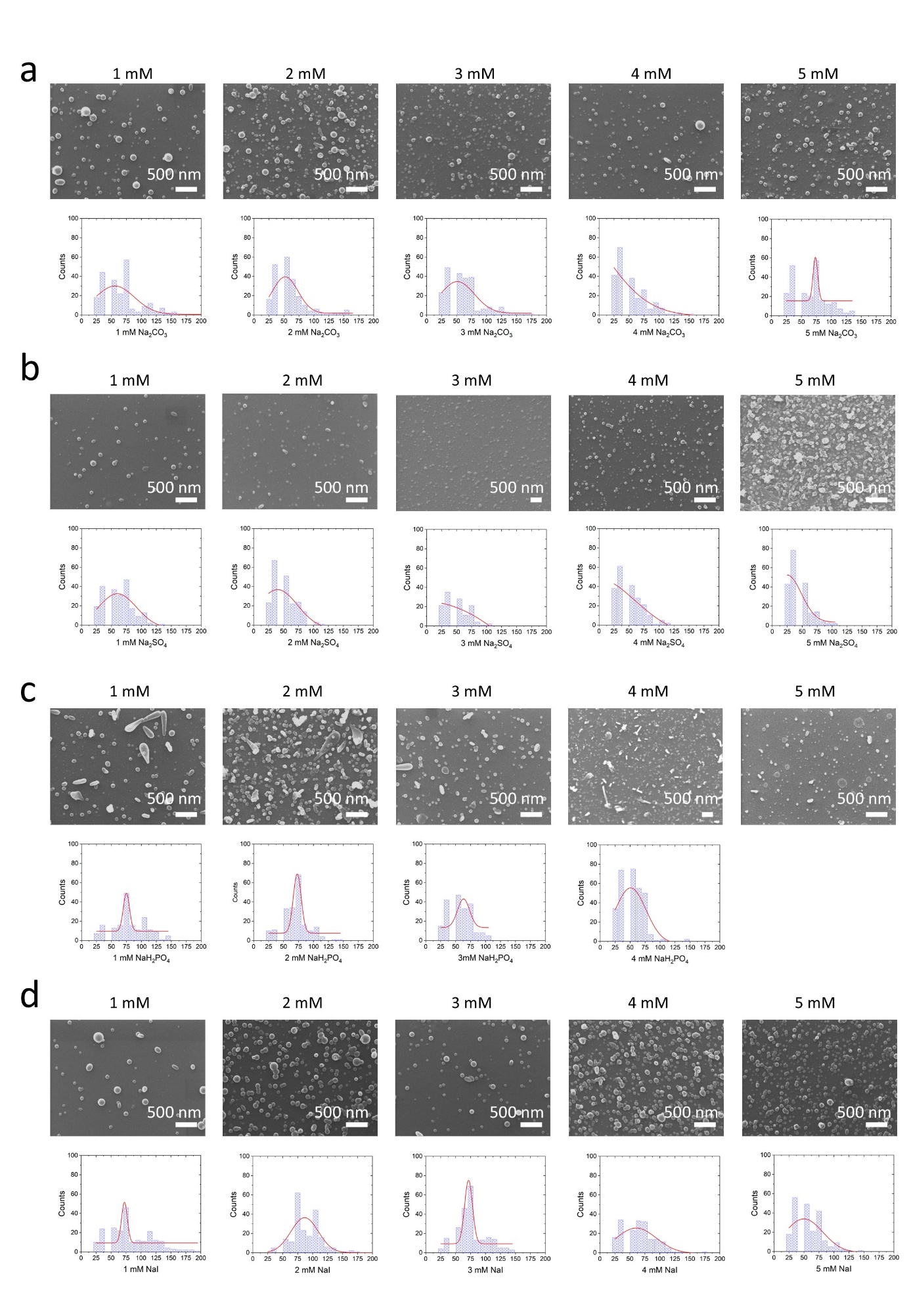


**Figure S7:** **Hofmeister anions modulate the size of HSA synthetic protein nanoparticles (SPNPs).** SEM images and corresponding size distribution histograms of as-jetted, non-crosslinked HSA SPNPs prepared from solutions containing varying concentrations of several Hofmeister series anions: (a) Na₂CO₃, (b) Na₂SO₄, (c) NaH₂PO₄, and (d) NaI. Scale bar: 500 nm.


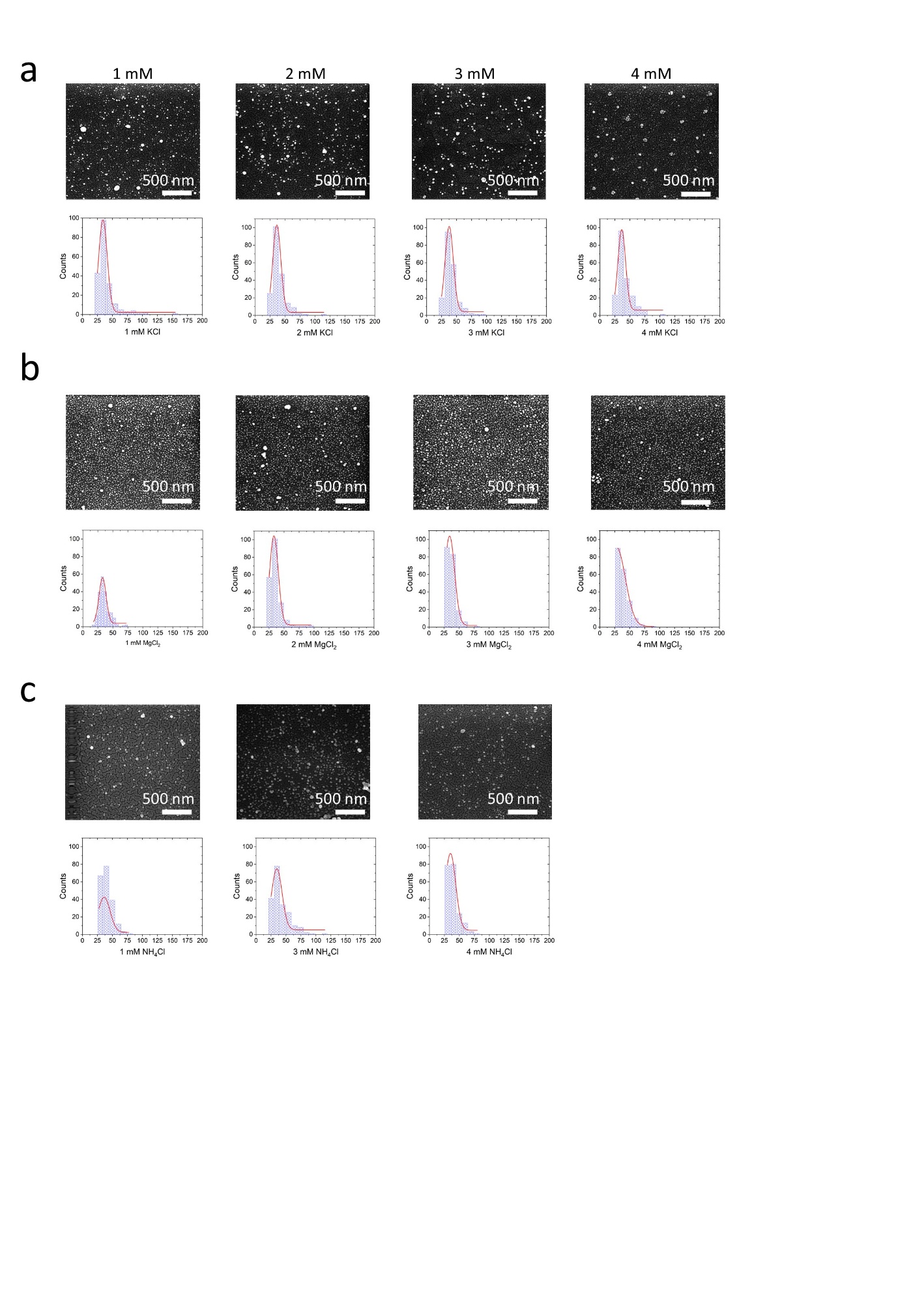


**Figure S8: Hofmeister cations affected the size of HSA synthetic protein nanoparticles (SPNPs).** SEM images and corresponding size distribution curves of as-jetted, non-crosslinked HSA SPNPs prepared from solutions with varying concentrations of several Hofmeister series cations: (a) NH₄Cl, (b) KCl, and (c) MgCl₂. Scale bar: 500 nm.


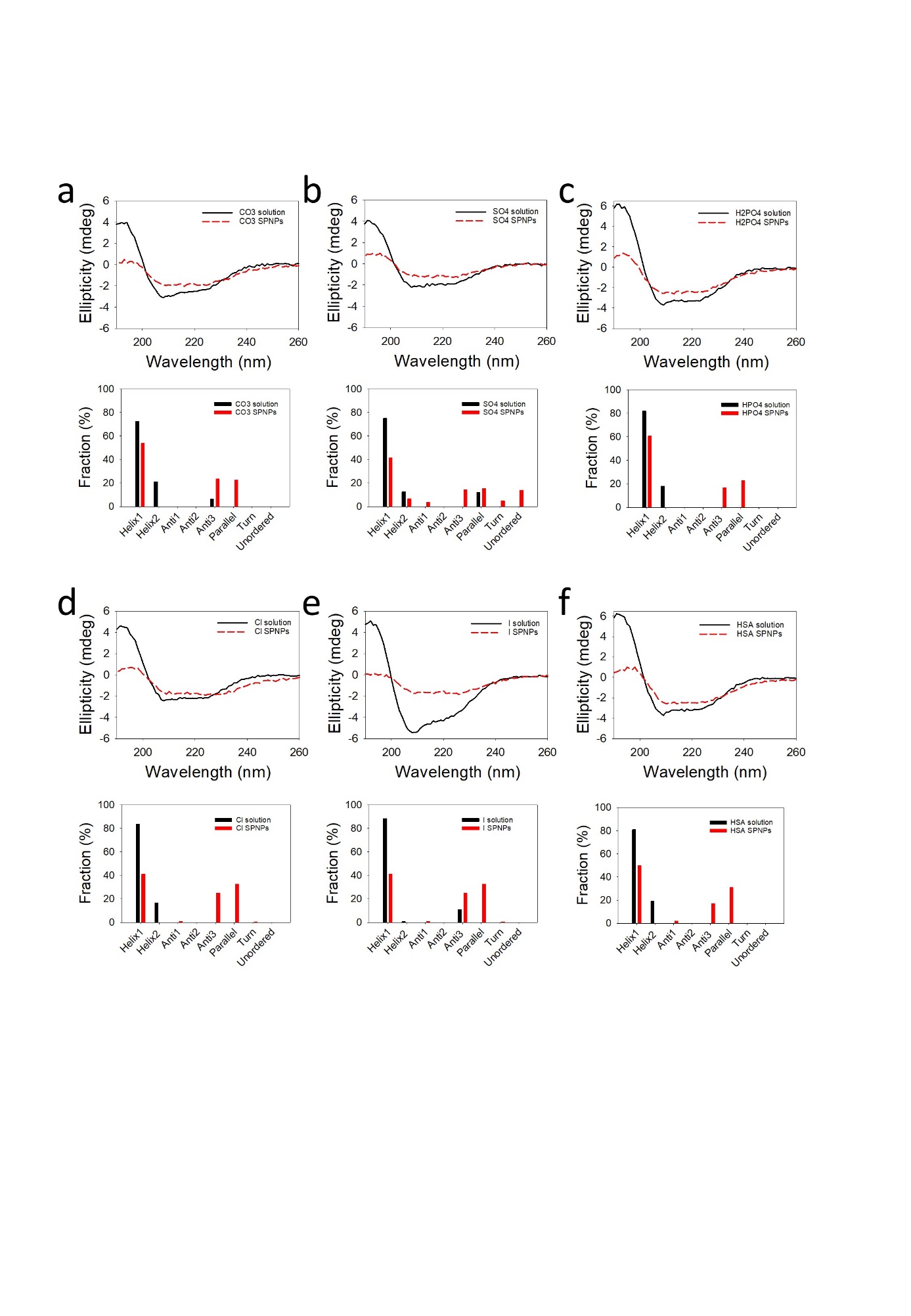


**Figure S9: Effect of Hofmeister anions on the secondary structure of proteins in HSA jetting solutions and their corresponding SPNPs.** Circular dichroism (CD) spectra of protein jetting solutions and the respective SPNPs suspended in ultrapure water, prepared from jetting solutions containing various Hofmeister anions: (a) Na₂CO₃, (b) Na₂SO₄, (c) NaH₂PO₄, (d) NaCl, (e) NaI, and (f) HSA without salt. Fractions of secondary structural elements derived from the CD spectra using BestSel analysis are also shown. Note: Due to strong UV absorption by iodide, CD analysis of free HSA in 2 mM NaI was performed after dilution to ~8 µM to obtain a readable spectrum.


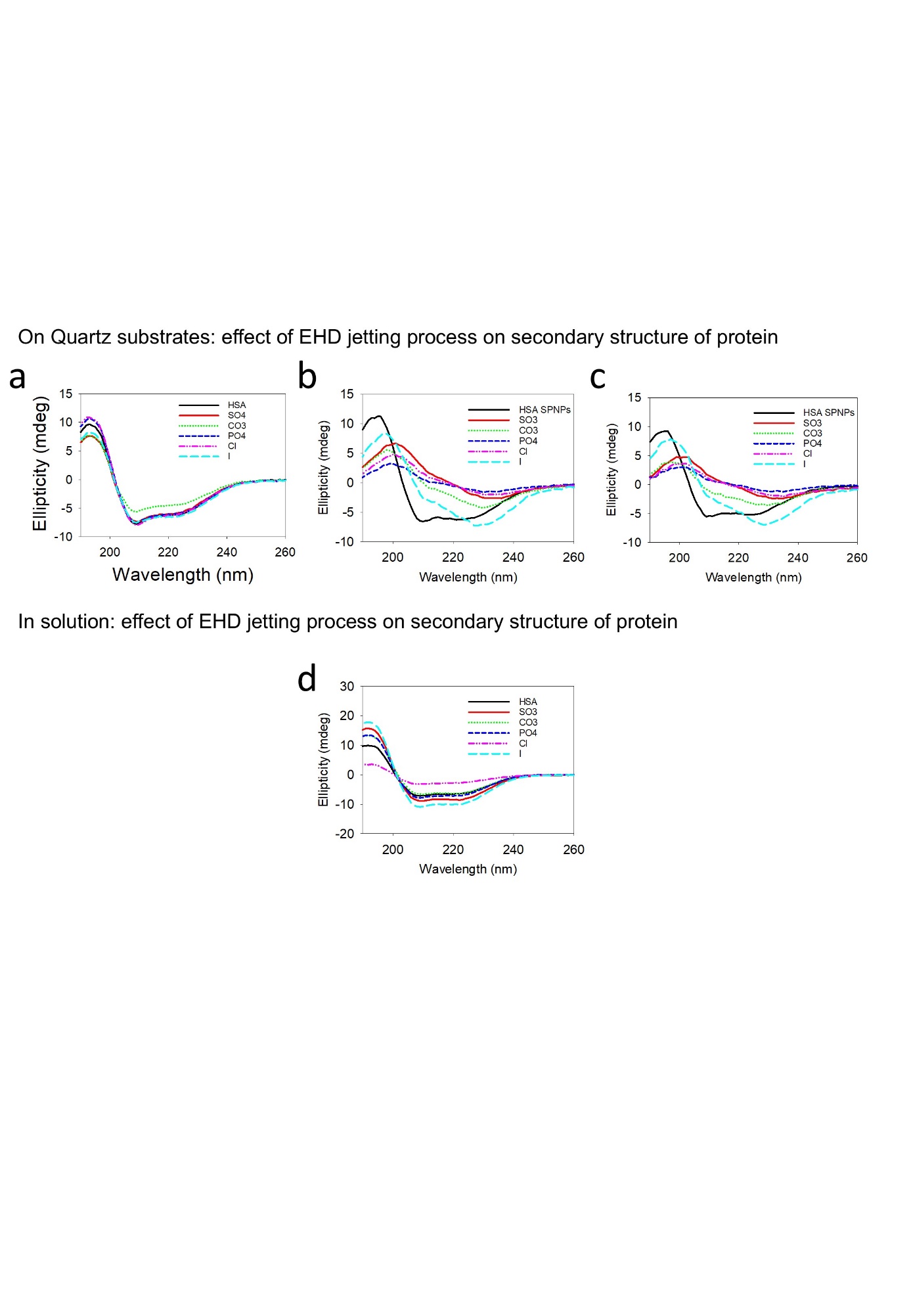


**Figure S10:** **Effect of Hofmeister anions and the EHD jetting process on protein secondary structure.** Circular dichroism (CD) spectra of (a) protein jetting solutions drop-cast on quartz substrates, (b) dry-state uncrosslinked SPNPs as-jetted on quartz, (c) dry-state crosslinked SPNPs as-jetted on quartz, and (d) as-jetted dry-state uncrosslinked SPNPs redissolved in ultrapure water. Spectra correspond to samples prepared with various Hofmeister anions, including Na₂SO₄, Na₂CO₃, NaH₂PO₄, NaCl, NaI, and HSA without salt.


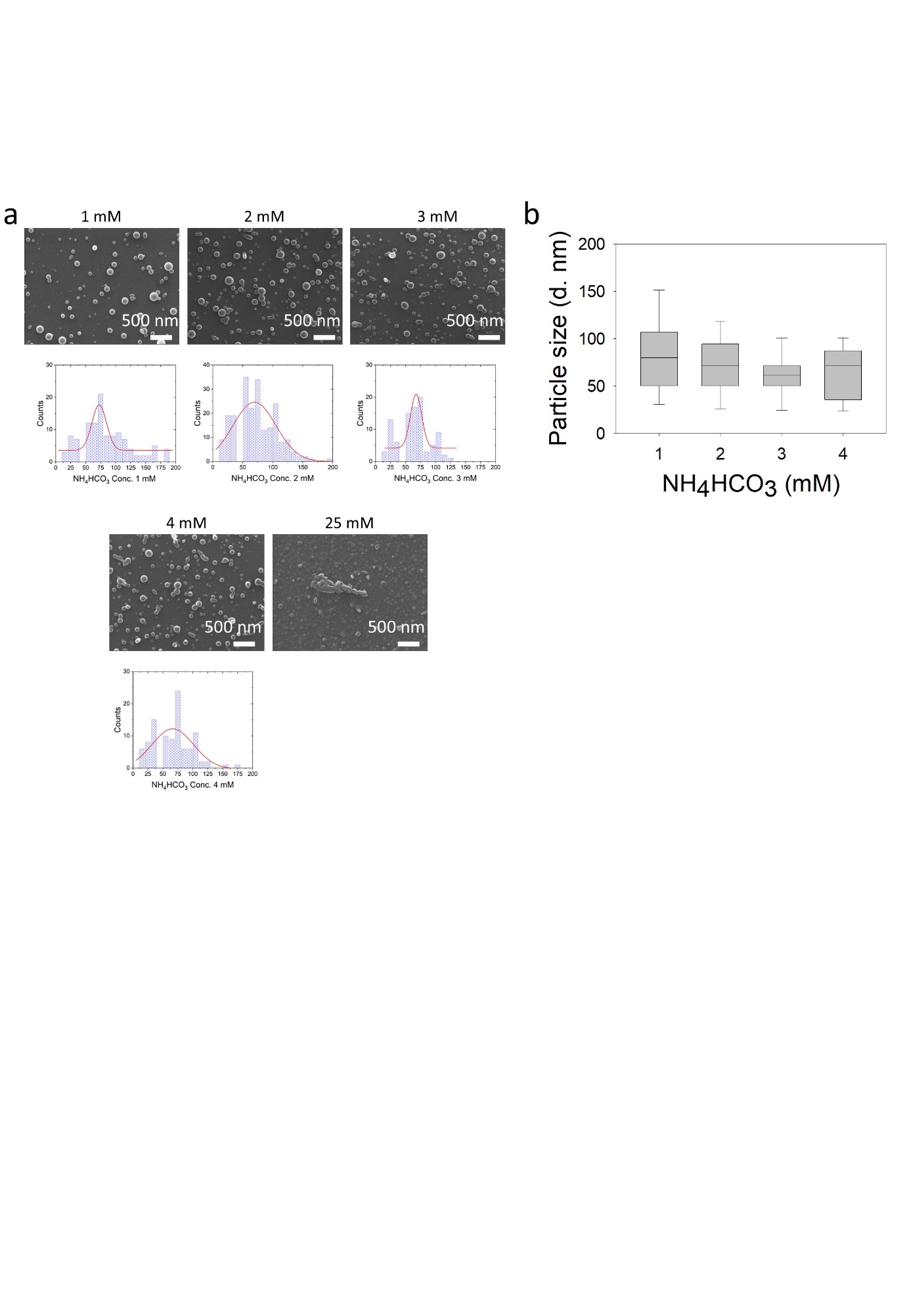


**Figure S11: Salt does not dictate the shape of the HSA synthetic protein nanoparticles (SPNPs). (a)** SEM images and corresponding size distribution curves of as-jetted, non-crosslinked SPNPs prepared with varying concentrations of NH₄HCO₃. Scale: 500 nm. (b) The plot of average SPNP diameter as a function of increasing salt concentration is also shown, with particle sizes calculated from SEM images (n = 200 nanoparticles per condition).


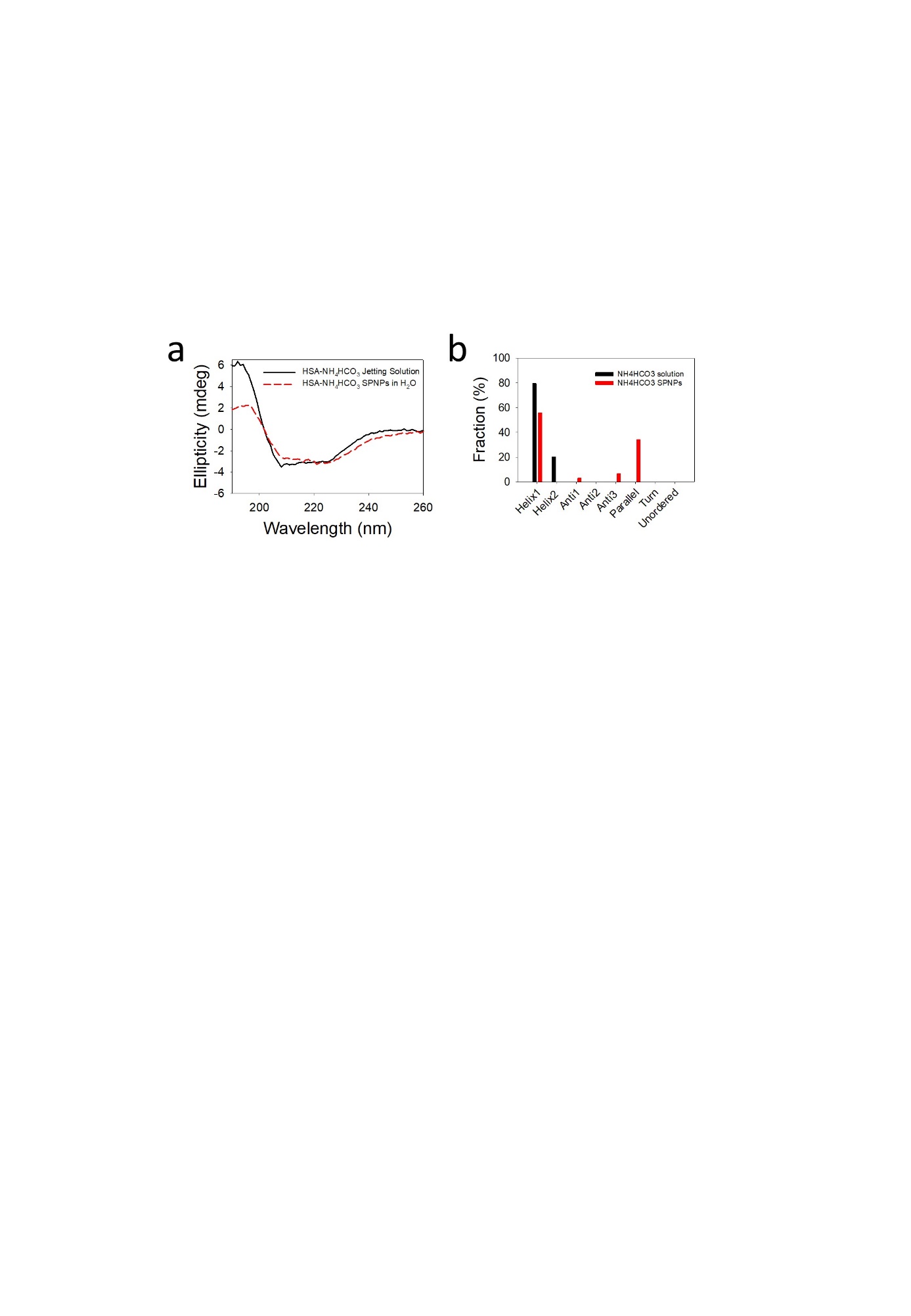


**Figure S12: Effect of ammonium bicarbonate on the secondary structure of proteins and their respective SPNPs.** (a) Circular dichroism (CD) spectra of protein jetting solutions and corresponding SPNPs suspended in ultrapure water. The HSA-NH₄HCO₃ solution refers to the jetting solution prepared by dissolving HSA in 20% v/v aqueous methanol containing 2 mM NH₄HCO₃. The HSA-NH₄HCO₃ SPNPs denote nanoparticles fabricated from the corresponding jetting solution. (b) Fractions of secondary structural elements derived from the CD spectra using BestSel analysis.
